# Impact of Wavelet Kernels on Predictive Capability of Radiomic Features: A Case Study on COVID-19 Chest X-ray Images

**DOI:** 10.3390/jimaging9020032

**Published:** 2023-01-30

**Authors:** Francesco Prinzi, Carmelo Militello, Vincenzo Conti, Salvatore Vitabile

**Affiliations:** 1 Department of Biomedicine, Neuroscience and Advanced Diagnostics (BiND), University of Palermo, 90127 Palermo, Italy; 2 Institute for High-Performance Computing and Networking, National Research Council (ICAR-CNR), 90146 Palermo, Italy; 3 Faculty of Engineering and Architecture, University Kore of Enna, 94100 Enna, Italy

**Keywords:** radiomic features, machine learning models, wavelet kernels, predictive capability, wavelet-derived features, chest X-ray images, COVID-19 prognosis

## Abstract

Radiomic analysis allows for the detection of imaging biomarkers supporting decision-making processes in clinical environments, from diagnosis to prognosis. Frequently, the original set of radiomic features is augmented by considering high-level features, such as wavelet transforms. However, several wavelets families (so called kernels) are able to generate different multi-resolution representations of the original image, and which of them produces more salient images is not yet clear. In this study, an in-depth analysis is performed by comparing different wavelet kernels and by evaluating their impact on predictive capabilities of radiomic models. A dataset composed of 1589 chest X-ray images was used for COVID-19 prognosis prediction as a case study. Random forest, support vector machine, and XGBoost were trained (on a subset of 1103 images) after a rigorous feature selection strategy to build-up the predictive models. Next, to evaluate the models generalization capability on unseen data, a test phase was performed (on a subset of 486 images). The experimental findings showed that *Bior1.5*, *Coif1*, *Haar*, and *Sym2* kernels guarantee better and similar performance for all three machine learning models considered. Support vector machine and random forest showed comparable performance, and they were better than XGBoost. Additionally, random forest proved to be the most stable model, ensuring an appropriate balance between sensitivity and specificity.

## 1. Introduction

Quantitative imaging biomarkers, i.e., radiomic features, can be used to extract information complementary to the visual approach of the radiologist [1]. Radiomics has been exploited in different scenarios to support the decision making of clinicians at different stages of the care process, from diagnosis to prognosis. With more details, radiomic signatures can be used for the diagnosis of several pathologies, to predict response to therapy, and to categorize clinical outcomes in general. Although several best practices [2] and standardization initiatives [3] have been proposed, full reproducibility of the radiomic process is still lacking. The imaging used, acquisition protocol, feature extraction setting, preprocessing, and the machine learning (ML) workflow are variables undermining the reproducibility of the radiomic process. Several researchers have tried to evaluate the robustness and the predictive capabilities of radiomic features, depending on specific external parameters (e.g., segmentation method, quantization level, and preprocessing steps) [4,5,6]. This already complex scenario becomes even more complicated when the classical radiomic feature set is extended by also considering high-level features, such as wavelet transforms, Laplacian filters (LoG), and intensity transformation (e.g., logarithm, exponential, gradient, etc.).

Wavelet-derived features showed strong predictive capabilities in several contexts: tumor-type prediction of early stage lung nodules in CT [7], neoadjuvant chemotherapy treatment prediction for breast cancer in MRI [8], low-dose rate radiotherapy treatment response prediction of gastric carcinoma in CT [9], liver cirrhosis detection [10], glioblastoma multiforme differentiation from brain metastases in MRI [11], and grading of COVID-19 pulmonary lesions in CT [12]. The wavelet-derived features are calculated on the image decompositions—four for 2D images (e.g., X-ray, mammography, ultrasound, etc.) and eight for 3D volumes (e.g., CT, MRI, etc.)—providing multi-resolution images. This leads to a substantial increase in the number of features that, if not properly managed, can bring predictive systems to incur the *curse of dimensionality* [13]. In addition, several families of wavelet transforms exist, some improved for noise reduction, others for image compression; however, in general, all provide a multi-resolution representation of the initial image. Considering that the predictive ability of wavelet-derived features overcomes the original ones, analyzing and comparing the behavior of wavelet kernels are worthwhile to provide a recommendation for their use.

In radiomics, wavelets are often used without paying attention to the type of kernel involved: the commonly followed approach is to use the default kernel for feature extraction, without evaluating *a-priori* which is more suitable for the specific clinical scenario. Few researchers have approached this problem. In [14], wavelet kernels were compared to evaluate the role of the CT radiomic features for lung cancer prediction: In [15], a similar approach was used for the diagnosis of colorectal cancer patients in contrast-enhanced CT. Both studies used CT imaging, a modality that can provide images with higher resolution and more defined details, compared with CXR projective imaging.

In this study an in-depth analysis is performed by comparing different wavelet kernels and by evaluating their impact on predictive capabilities of radiomic models. As case study, three machine learning radiomics models were implemented to predict the prognosis of COVID-19 patients from chest X-ray (CXR) images. The *Biorthogonal*, *Coiflets*, *Daubechies*, *Discrete Meyer*, *Haar*, *Reverse Biorthogonal*, and *Symlets* wavelet families were considered to quantify and compare the predictive performance of the radiomic features. The radiomic features were extracted from the decomposed images, preprocessed, selected, and then used to train several ML models, including random forest (RF), XGBoost (XGB), and support vector machine (SVM). The models and the different wavelets were compared to evaluate the most predictive kernel.

The remainder of this paper is structured as follows: Section 2 describes the dataset used, the extraction and preprocessing of radiomic features, and the model training. Section 3 provides the results obtained for the wavelet kernels and trained models. Section 4 discuss the experimental findings. Finally, Section 5 outlines the study conclusions.

## 2. Materials and Methods

### 2.1. Dataset Characteristics and Lung Delineation

The dataset used is composed of 1589 CXR images of COVID-19 patients, labeled as ’SEVERE’ and ’MILD’ disease, according to [16]. This dataset is split into 1103 and 486 patients used for the training and test phases, respectively. This partition was established by the organizing committee of the COVID CXR Hackathon competition [17], who made these datasets available. In more detail, the training dataset (1103 samples) include 568 severe and 535 mild; the test dataset (486 samples) include 180 severe and 306 mild. From a visual evaluation, it was possible to note that the multicentric dataset (collected by six different hospitals) is heterogeneous in terms of image size, quality, gray levels distribution, and origin (some are native digital images, whereas others are obtained by scanning traditional X-ray films). In particular, regarding the size: for the training dataset, the most frequent size is 2336×2836 pixels (35.7%); the other images have variable sizes (1396−4280×1676−4280 pixels). For the test set (composed of 486 images), 88.27% of the images have 2336×2836 pixels; the others have variable sizes (1648−3027×1440−3000 pixels). According to [16], in our study, all the images were resized at 1024×1024. The CXR images were stored in .PNG format with a 96 DPI resolution (pixel size ≃0.265×0.265 mm), and no metadata related to acquisition details are available.

**Figure 1:**
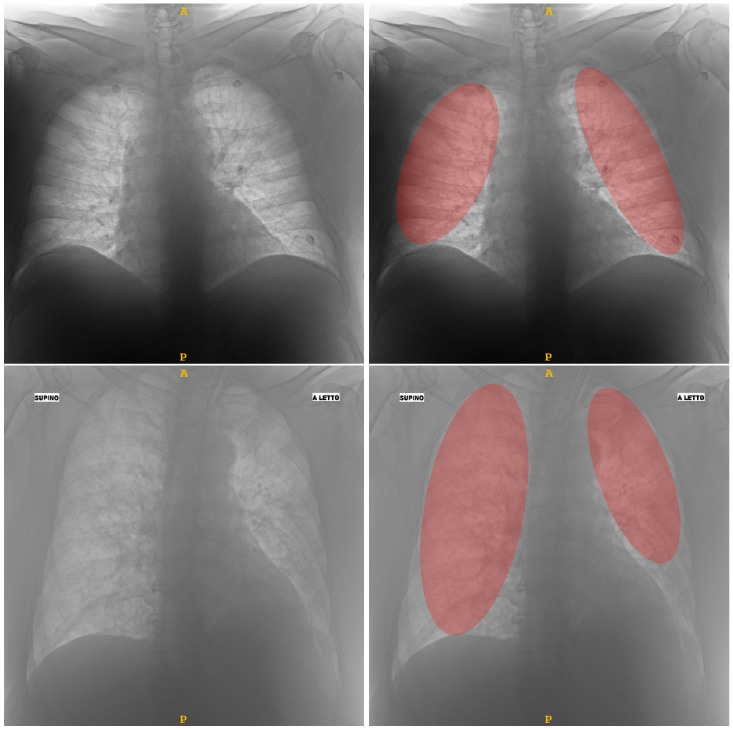
Two examples of segmentation obtained by means of the semi-automatic tool able to assist clinician to detect the elliptical ROIs (highlighted in red) within lungs: in the upper row a MILD sample; in the lower row a SEVERE sample.

To extract the radiomic features, the regions of interest (ROIs) containing the lungs have to be identified. To achieve this, a MATLAB^®^-coded custom tool was implemented to delineate the lung ROIs: in particular, a semi-automatic delineation modality for the identifying of the maximum elliptical ROI contained within the lung was implemented. This choice was motivated by the need to focus the attention on the central region of the lung, excluding peripheral zones. In Figure 1 two segmentation examples. Due to the excessive heterogeneity of the CXR dataset, automatic lung segmentation approaches did not provide satisfactory results. For this reason, it was decided to implement a tool that can easily support clinicians during the image annotation procedure. Specifically, the implemented semi-automatic algorithm allows the automatic identification of the bounding-box containing the lung and determining the maximum elliptical ROI contained within it. The clinician can decide whether to accept it, if the result is satisfactory, or to modify it to find a more suitable fitting between the elliptic ROI and the lung area by changing orientation and size of the ellipse. Because this is a supervised semi-automatic approach (each segmentation is directly validated), and considering the experience of the clinicians who supported this study, no evaluation step was performed. We decided to implement an *ad hoc* computer-assisted tool that can simply and intuitively guide clinicians through the steps of lung segmentation, from CXR image selection to segmentation mask storage.

### 2.2. Wavelet Transform

The widespread use of wavelet transforms—used in several applications concerning signal and image processing—is due to their ability to capture information in both the frequency and time domains. In this study, discrete wavelet transform (DWT) was applied to CXR images. The image passed through high-pass $h_{\psi }$ and low-pass $h_{\phi }$ filtering operations, decomposing the images into high-frequency (details) and low-frequency components (approximation). The decomposition of the image into subimages at different resolution allows for multi-resolution analysis [18,19]. For this reason, DWT has found numerous applications in image processing, focusing on denoising [20] and compression [21,22]. DWT is computed through two functions, the *scaling function* and the *wavelet function*[23]. For a 2D signal f(x,y) of size M×N (such as the CXR images considered in this study), the DWT is defined as:

(1) $$W_{\phi }\left(j_{o},m,n\right)=\frac{1}{\sqrt{MN}}\sum_{x=0}^{M-1}\sum_{y=0}^{N-1}f\left(x,y\right)\phi_{j_{o,m,n}}\left(x,y\right)$$

(2) $$W_{\psi }^{I}\left(j,m,n\right)=\frac{1}{\sqrt{MN}}\sum_{x=0}^{M-1}\sum_{y=0}^{N-1}f\left(x,y\right)\psi_{j,m,n}^{I}\left(x,y\right)$$

where:

- Equation (1) represents the scaled version of the image, computed with the scaling function defined in Equation (3);
- Equation (2) defines the horizontal (*H*), vertical (*V*), and diagonal (*D*) representation of image (I={H,V,D}), computed with the wavelet function defined in Equation (4)).

(3) $$\;\phi_{j_{o,m,n}}\left(x,y\right)=2^{j/2}\phi \left(2^{j}x-m,2^{j}y-n\right)$$

(4) $$\psi_{j,m,n}^{I}\left(x,y\right)=2^{j/2}\psi \left(2^{j}x-m,2^{j}y-n\right)$$

Finally, the combined application of the *scaling function* (ϕ) and the *wavelet function* (ψ) obtains four image decompositions (LL, LH, HL, and HH) for 2D transforms, as indicated in Equations (5)–(8). The same formulas can be adapted and extended for the 3D case, to consider volumetric imaging (e.g., MRI, CT, etc.). DWT then depends on the low-pass $h_{\phi }$ and high-pass $h_{\psi }$ kernel chosen for decomposition.

(5) LL=ϕ(x,y)=ϕ(x)ϕ(y)

(6) $$\;LH=\psi^{H}\left(x,y\right)=\psi \left(x\right)\phi \left(y\right)$$

(7) $$\;HL=\psi^{V}\left(x,y\right)=\phi \left(x\right)\psi \left(y\right)$$

(8) $$HH=\psi^{D}\left(x,y\right)=\psi \left(x\right)\psi \left(y\right)$$

In this study the *Biorthogonal* (Bior1.5), *Coiflets* (Coif1), *Daubechies* (Db3), *Discrete Meyer* (Dmey), *Haar*, *Reverse Biorthogonal* (Rbio1.5), and *Symlets* (Sym2) wavelet families [24] were considered. The following are the main applications of wavelet families:

- *Biorthogonal*: commonly used for denoising, in particular when white Gaussian noise is present [25];
- *Reverse Biorthogonal*: used for compression [26] and denoising [27];
- *Coiflet*: used for compression [28] and denoising [29];
- *Daubechies*: provides excellent performance in compression and are popular choice in medical imaging applications [30];
- *Discrete Meyer*: in general used for multi-resolution analysis [31] and some variants for edge and blocking artifact reduction [32];
- *Haar*: is the first introduced and several generalizations and modifications were proposed [33]. It is one of the most widely used and has many medical imaging applications, including image fusion [34] and compression in radiography [35], CT, and MRI [36];
- *Symlets*: is a modified version of *Daubechies* wavelets with increased symmetry [37], used for signal decomposition including characterization of fabric texture [38].

As each wavelet family consists of several kernels, and they were experimentally selected to qualitatively and visually maintain decomposed images similar to the original image. Table 1 summarizes the chosen kernels and the respective number of coefficients. Except for *Dmey*, kernels with a number of coefficients less than or equal to 10 were chosen.

### 2.3. Radiomic Features Extraction

The radiomic features were extracted from CXR images using PyRadiomics [39] and segmentation masks were obtained through the semi-automated tool described in Section 2.1. The extracted radiomic features belong to the following six categories:

- First order (FO) intensity histogram statistics;
- Gray level co-occurrence matrix (GLCM) [40,41];
- Gray level run length matrix (GLRLM) [42];
- Gray level size zone matrix (GLSZM) [43];
- Gray level dependence matrix (GLDM) [44];
- Neighboring gray tone difference matrix (NGTDM) [45].

Moreover, each ROI was filtered considering all families of the wavelet transforms discussed above. Specifically, for each image and for each wavelet family, four decompositions (LL, LH, HL, and HH) were calculated, and then the features were extracted by obtaining a total of 93×4=372 features. Finally, the original radiomic features (without wavelet filtering) were also extracted to compare the wavelet-derived vs. original predictive capabilities.

### 2.4. Radiomic Features Preprocessing

To obtain a subset of non-redundant features with relevant information content, preprocessing and selection were performed with the following steps [2]:

- **Near-zero variance analysis**: aimed at removing features with low information content. This operation considered a variance cutoff of 0.01: features with a variance less than or equal to this threshold were discarded;
- **Correlation analysis**: aimed at removing highly correlated features, by means of the Spearman correlation for pairwise feature comparison. For each set of N correlated features, N-1 were removed. Specifically, the correlation matrix was first calculated, and then it was analyzed according to the following decomposition priority: LH, HL, HH, and LL. As values larger than 0.80 are commonly used for Spearman correlation [46,47,48,49], and a threshold of 0.85 was chosen.
- **Statistical analysis**: the Mann–Whitney U test was used to test the difference between mild and severe distribution, computing the *p*-value for each features selected from the previous step. The *p*-value threshold was set to 0.05.

### 2.5. Features Selection and Model Training

To select the most discriminating features, the sequential feature selector (SFS) [50] algorithm was used. The sequential feature selector was set in *forward mode*, which, at each step, includes a feature, and in *floating mode*, which, after the inclusion, performs the exclusion, allowing us to consider more feature subset combinations. This allowed for the selection of the best radiomic signature for each model considered (i.e., RF, SVM, and XGB). The SFS algorithm was applied considering a 10-fold stratified cross validation (CV) and using all the selected features in the preprocessing step.

The experiments were conducted in the Python 3.7 environment, using the scikit-learn 1.0.2 and xgboost 1.2.1 libraries for model training. In particular:

- RF was trained using the bootstrap technique with 100 estimators and the Gini criterion.
- SVM was trained setting the regularization parameter C=1.0, considering the radial basis function as kernel, the coefficient $\gamma =1/(n_{features}\times \sigma )$, the shrinking method [51], and the probability estimates to enable the AUROC computation. In addition, for SVM, the features were standardized before the training.
- XGB was trained using 100 estimators, $depth_{max}=6$, and ‘gain’ as the importance type. In addition, the binary logistic loss function was used to model the binary classification problem, considering a learning rate of η=0.3.

Accuracy, sensitivity, specificity, and AUROC were calculated for all the three ML models and all the wavelet kernels considered. In addition, for a precise estimation of the trained models, in the training phase, performance was calculated by considering a stratified CV, repeated 20 times on the 1103 samples dataset. Successively, to evaluate the models generalization capability on unseen data, a test phase was performed on the 486 samples dataset.

## 3. Results

### 3.1. Features Preprocessing

**Figure 2:**
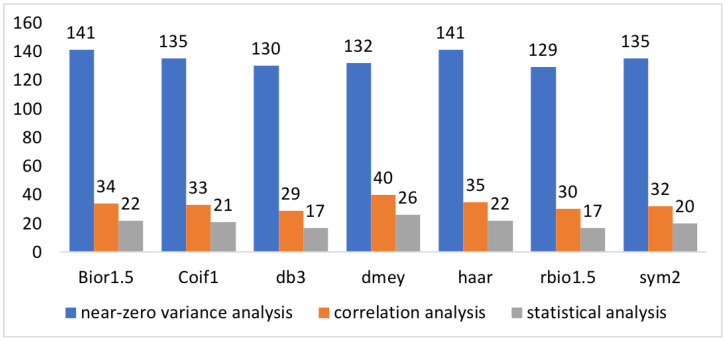

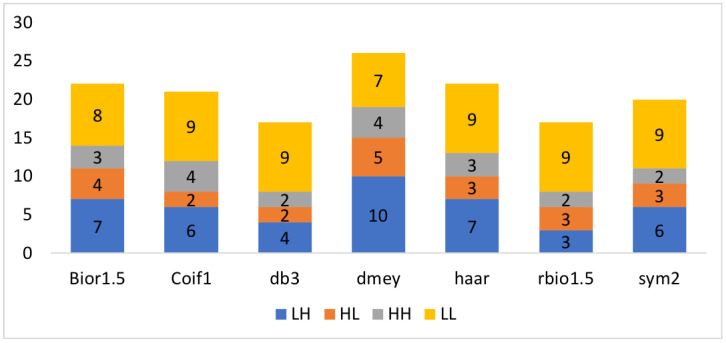
**Upper**: Selected features after near-zero variance analysis (in blue), correlation analysis (in orange), and statistical analysis (in gray), respectively. **Lower**: Selected features considering each wavelet decomposition.

Figure 2 shows the selected radiomic features after each preprocessing step (upper), considering each wavelet decomposition (lower). After near-zero variance analysis, *Dmey*, *Bior1.5*, and *Haar* kernels had the most features selected, while, at the end of preprocessing, the number of features was comparable for each kernel used. As expected, a marked overlap between the selected features belonging to the LL decomposition was observed (Table 2). This finding resulted from the nature of the LL decomposition, which is essentially derived from a resizing. For the other decompositions (LH, HL, and HH), this overlap decreased because the application of the kernel corrupted the image in different ways among the various wavelet families. For each kernel, the complete list of radiomic features remaining after the preprocessing phase is reported in Table 2.

### 3.2. Features Selection

The radiomic features remaining at the end of the preprocessing phase represented the input of the SFS wrapper method, which were used to select the most discriminating radiomic signature for the SVM, RF, and XGB models. The number of features maximizing accuracy was selected. For equal accuracy among the different radiomic signatures, the one with the lower standard deviation was selected. The features selection stage allows us to select only the discriminating features: in fact, considering all available features (at the end of preprocessing) can lead to a performance degradation. Table 3 summarizes the number of features for each wavelet kernel and ML model used for training. On average, a signature consisting of 12–15 features was selected. Additionally, overall, the decision tree-based models (RF and XGB) performed better, with more features than SVM. The details of the features selected for each wavelet kernel and for each ML model are reported in Appendix A (Table A1, Table A2, Table A3).

### 3.3. Predictive Model Results

Table 4, Table 5, Table 6 and Table 7 show the accuracy, sensitivity, specificity, and AUROC obtained in the experimental trials, both in the training and testing phases. The reported metrics obtained in the training are represented as *mean* ± *standard deviation*, because the averaged values calculated considering 20 repetitions of the 10-fold stratified CV.

To verify whether the values obtained by CV repetitions are statistically different, the ANOVA test was used: for each model (i.e., XGB, SVM, and RF), the accuracy values obtained for each fold were compared, considering the used kernels as groups, obtaining the *p*-values << 0.05.

Focusing on wavelet kernels, *Db3*, *Dmey*, and *Rbio1.5* proved to be the worst for all three ML models. This assessment resulted from the high imbalance between sensitivity and specificity, suggesting overfitted models (high sensitivity vs. low specificity or vice versa). The AUROC is the most widely used index of global diagnostic accuracy, since higher values correspond to a better selective ability of the biomarkers [52]. Excluding *Db3*, *Dmey*, and *Rbio1.5* for their unbalanced performance, overlapping AUROC values were obtained in tests for *Bior1.5*, *Coif1*, *Haar*, and *Sym2* kernels.

Focusing on ML models, XGB was the least accurate model, compared with SVM and RF, which showed comparable accuracy on the test set. Moreover, RF, considering the smaller gap in training and testing performance, had a strong generalization ability. In summary, wavelet-derived features were more predictive than the original ones. This result was confirmed for all three ML models employed.

**Figure 3:**
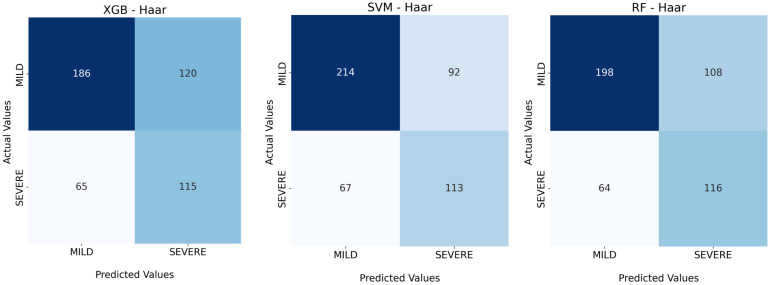
The confusion matrices of the three ML classifiers obtained with the *Haar* kernel.

Figure 3, shows the confusion matrices obtained by the three ML classifiers considering *Haar* as the wavelet kernel.

## 4. Discussion

Radiomic features can support the radiologists by providing a quantitative viewpoint that is complementary to the human visual perspective. Although many researchers have used classical radiomic features (i.e., FO, GLCM, GLRLM, GLSZM, and GLDM), many others have achieved increased predictive power by exploiting high-level features, computed from filtered images by means of wavelet transforms, LoG filters, and intensity transformation. In particular, the wavelet-derived features have demonstrated their predictive capability in several contexts [7,8,9,11,12]. Despite their widespread use in the literature, comparing the different wavelets kernels is difficult because of the few studies [14,15] focused on the problem. Consequently, which wavelet kernel has stronger discriminatory power is unclear.

**Figure 4:**
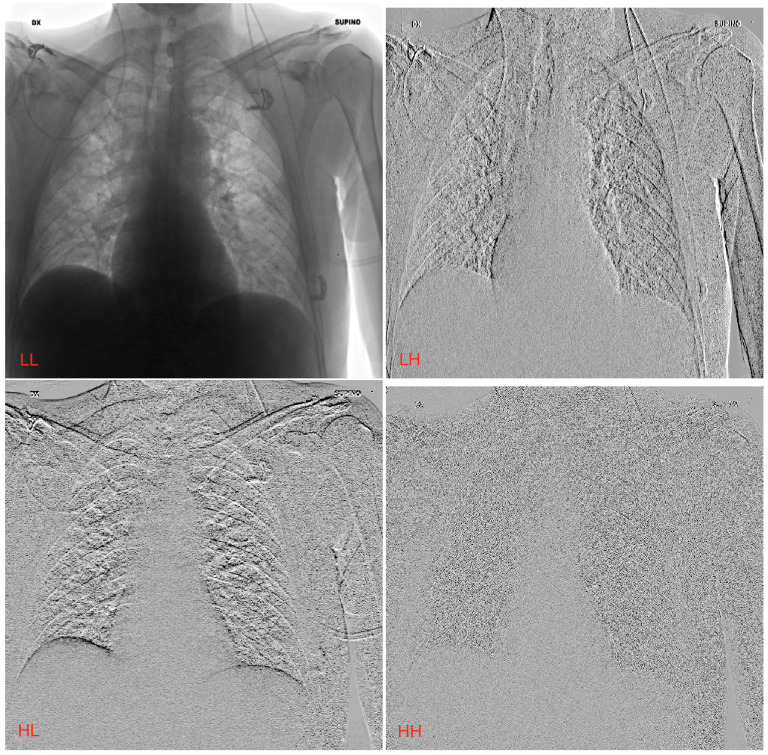
The four decompositions (LL, LH, HL, and HH) calculated by means of wavelets. In the example, the *Haar* kernel was used.

In this study, a CXR dataset was used to evaluate the impact of wavelet kernels on the radiomic model performance for predicting COVID-19 prognosis. Despite their projective nature, CXR images (consisting of less detailed images than volumetric CT series) are crucial to sustainably (e.g., cheaply and quickly) support healthcare systems. In this context, the ability of wavelets to provide multi-resolution imaging can be used to improve the predictive capabilities of radiographic imaging. *Bior1.5*, *Coif1*, *Haar*, and *Sym2* kernels were the best, in terms of the predictivity for all three ML models used (i.e., XGB, SVM, and RF). Conversely, *Db3*, *Dmey*, and *Rbio1.5* showed a serious imbalance between sensitivity and specificity, suggesting overfitted models. Finally, RF showed the strongest generalization ability, demonstrating less performance degradation between the training and testing phases. Figure 4 shows an example of the *Haar* transform in the four decompositions (LL, LH, HL, and HH). LL represents the rescaled image, which retains features approximating the original image. For the other decompositions (LH, HL, and HH), however, the image shows completely different characteristics, especially in the lung regions. The results obtained encourage the use of higher-level features, such as those obtained by wavelet transforms. The models trained with wavelet-derived features outperformed the model trained with original features. This is the proof that the ability to provide a multi-resolution image representation improves the prediction performance of the ML models. The findings of this study, applied to the prognosis of COVID-19, are a starting point for other pathologies analyzed by radiographic imaging. However, further analysis is required when volumetric imaging (e.g., CT and MRI) is used, in which the wavelet transform involves three spatial components.

## 5. Conclusions

Wavelet transforms represent a powerful tool for extracting biomarkers in radiomics. This study showed how the correct choice of the wavelet kernel used for the filtering and extraction of radiomic features improves the classification process, with respect to the use of the original features. At present, the discussion between shallow learning and deep learning is still open, especially in clinical scenarios, where the amount of available samples is not sufficient to train and use deep architectures. In this context, traditional ML techniques are also experiencing a second wave, in relation to the increased possibility of obtaining interpretable features and explainable models. In addition, traditional ML does not require a large amount of data to train the model.

Wavelet-derived features must be used wisely. In fact, having a reduced dataset limits the number of features that can be extracted and used to avoid incurring the *`curse of dimensionality’* [13]. This phenomenon shows that, with a fixed number of training samples, the average (expected) predictive power of a classifier improves as the dimension and the number of used features increase. This is due to the model’s overfitting on high-dimensionality and redundant data, which leads to improved performance on train data, but reduces the generalization capabilities on unseen test data. Although the study mainly focuses on evaluating how predictivity impacts the wavelet kernel changes, the interpretability of radiomic features must also be studied. In clinical contexts, explainable models are crucial, so that the models can be clinically validated and compared with the medical literature. Explainability improves the usability and acceptability of artificial intelligence (AI) models, as it allows the users to be involved in the debugging and model building processes [53]. In many intensive decision-based tasks, the interpretability of an AI-based system may emerge as an indispensable feature [54]. However, radiomic features have the disadvantages of being low-level, less abstract than deep features, and less informative, which can degrade model performance. This phenomenon can be mitigated by considering higher-level radiomic features, such as wavelet-derived ones, where the interpretability is sacrificed for the benefit of performance. If correlating the quantitative value with clinical meaning is easy with the original radiomic features, for wavelet-derived features, this task becomes more complicated because the physician does not use a wavelet transform in regular activities. However, their use achieves a trade-off between performance and interpretability.

## Figures and Tables

**Table 1:** Number of coefficients that define the kernel length.

Wavelet Kernel	Coefficients Number
Bior1.5	10
Coif1	6
Db3	6
Dmey	62
Haar	2
Rbio1.5	10
Sym2	4

**Table 2:** Complete lists of radiomic features remaining after the preprocessing step for each of the wavelet kernels considered.

Wavelet	Radiomic	Feature	Wavelet Kernel						
Decomposition	Category	Name	Bior1.5	Coif1	Db3	Dmey	Haar	Rbio1.5	Sym2
LL	FO	10Percentile	X	X	X	X	X	X	X
LL	FO	90Percentile	X	X	X	X	X	X	X
LL	FO	Kurtosis	X	X	X	X	X	X	X
LL	FO	Minimum	X	X	X	X	X	X	X
LL	FO	Range	X	X	X	X	X	X	X
LL	FO	Skewness	X	X	X	X	X	X	X
LL	GLRLM	GrayLevelNonUniformity		X	X		X	X	X
LL	GLSZM	HighGrayLevelZoneEmphasis	X	X	X	X	X	X	X
LL	GLSZM	SmallAreaHighGrayLevelEmphasis	X	X	X		X	X	X
**Decomposition**	**Category**	**Name**	**Bior1.5**	**Coif1**	**Db3**	**Dmey**	**Haar**	**Rbio1.5**	**Sym2**
LH	FO	Energy	X	X			X		X
LH	FO	Kurtosis	X				X		
LH	FO	Skewness	X	X			X		X
LH	GLRLM	LongRunEmphasis			X	X		X	
LH	GLSZM	GrayLevelNonUniformity				X			
LH	GLSZM	HighGrayLevelZoneEmphasis	X			X	X		
LH	GLSZM	LargeAreaEmphasis				X			
LH	GLSZM	SizeZoneNonUniformity				X			
LH	GLSZM	SmallAreaHighGrayLevelEmphasis				X			
LH	GLSZM	ZoneEntropy	X	X	X	X	X		X
LH	GLDM	DependenceNonUniformity	X	X	X	X	X	X	X
LH	GLDM	DependenceVariance		X					X
LH	GLDM	LargeDependenceEmphasis	X	X	X	X	X	X	X
LH	GLDM	LargeDependenceHighGrayLevelEmphasis				X			
HL	FO	Kurtosis	X				X		
HL	FO	Skewness		X					X
HL	FO	Maximum				X		X	
HL	GLRLM	LongRunEmphasis			X	X		X	
HL	GLSZM	HighGrayLevelZoneEmphasis	X						
HL	GLSZM	LargeAreaEmphasis	X				X		
HL	GLSZM	SizeZoneNonUniformity				X			
HL	GLSZM	SmallAreaHighGrayLevelEmphasis				X			
HL	GLSZM	ZoneEntropy	X	X	X	X	X	X	X
HL	GLDM	DependenceVariance							X
HH	FO	Minimum		X					X
HH	FO	Skewness		X					
HH	FO	Range				X			
HH	GLRLM	LongRunEmphasis							X
HH	GLRLM	LongRunHighGrayLevelEmphasis		X	X				
HH	GLSZM	GrayLevelNonUniformity	X		X	X	X	X	
HH	GLSZM	HighGrayLevelZoneEmphasis	X				X		
HH	GLSZM	SizeZoneNonUniformity				X		X	
HH	GLSZM	ZoneEntropy	X	X			X		
HH	GLDM	LargeDependenceHighGrayLevelEmphasis				X			
TOTAL SELECTED FEATURES			22	21	17	26	22	17	20

**Table 3:** Features selected using the SFS, for each wavelet kernel with the ML models considered.

Wavelet Kernel	Initial Features (After Preprocessing)	Machine Learning Model		
		XGB	SVM	RF
Bior1.5	22	17	14	15
Coif1	21	15	9	13
Db3	17	10	10	13
Dmey	26	14	15	12
Haar	22	19	15	16
Rbio1.5	17	13	13	10
Sym2	20	16	8	12
no wavelet	11	8	9	9

**Table 4:** Accuracy values obtained in training (*mean* ± *standard deviation*) and in testing phases for each wavelet kernel, with three ML models considered. Boldface values highlight the best three obtained results.

Wavelet Kernel	Machine Learning Model					
	XGB		SVM		RF	
	Train	Test	Train	Test	Train	Test
Bior1.5	0.633±0.041	0.604	0.671±0.041	0.641	0.661±0.043	0.646
Coif1	0.635±0.042	0.619	0.671±0.043	0.627	0.662±0.039	0.631
Db3	0.629±0.046	0.541	0.655±0.045	0.641	0.641±0.040	0.594
Dmey	0.633±0.047	0.555	0.654±0.044	0.578	0.624±0.043	0.592
Haar	0.654±0.045	0.619	0.683±0.046	0.673	0.674±0.044	0.646
Rbio1.5	0.627±0.047	0.586	0.646±0.047	0.606	0.647±0.045	0.600
Sym2	0.644±0.040	0.611	0.672±0.041	0.644	0.650±0.044	0.650
no wavelet	0.611±0.047	0.567	0.636±0.042	0.619	0.630±0.046	0.594

**Table 5:** Sensitivity values obtained in training (*mean* ± *standard deviation*) and in testing phases for each wavelet kernel, with three ML models considered. Boldface values highlight the best three obtained results.

Wavelet Kernel	Machine Learning Model					
	XGB		SVM		RF	
	Train	Test	Train	Test	Train	Test
Bior1.5	0.647±0.060	0.588	0.660±0.060	0.633	0.662±0.066	0.627
Coif1	0.646±0.064	0.622	0.671±0.066	0.627	0.655±0.062	0.616
Db3	0.639±0.070	0.577	0.643±0.058	0.683	0.642±0.059	0.627
Dmey	0.646±0.070	0.638	0.649±0.064	0.722	0.623±0.063	0.738
Haar	0.661±0.067	0.639	0.660±0.070	0.628	0.683±0.062	0.644
Rbio1.5	0.635±0.062	0.550	0.649±0.064	0.638	0.637±0.064	0.616
Sym2	0.651±0.061	0.644	0.649±0.063	0.594	0.652±0.063	0.611
no wavelet	0.619±0.066	0.655	0.614±0.065	0.683	0.621±0.068	0.622

**Table 6:** Specificity values obtained in training (*mean* ± *standard deviation*) and in testing phases for each wavelet kernel, with three ML models considered. Boldface values highlight the best three obtained results.

Wavelet Kernel	Machine Learning Model					
	XGB		SVM		RF	
	Train	Test	Train	Test	Train	Test
Bior1.5	0.618±0.061	0.614	0.683±0.062	0.647	0.661±0.061	0.656
Coif1	0.622±0.069	0.617	0.671±0.056	0.627	0.669±0.063	0.640
Db3	0.618±0.061	0.519	0.667±0.065	0.617	0.641±0.064	0.575
Dmey	0.618±0.064	0.506	0.658±0.062	0.493	0.625±0.063	0.506
Haar	0.646±0.067	0.608	0.709±0.065	0.699	0.665±0.066	0.647
Rbio1.5	0.618±0.065	0.607	0.643±0.066	0.588	0.657±0.060	0.591
Sym2	0.636±0.056	0.591	0.697±0.059	0.673	0.648±0.068	0.637
no wavelet	0.601±0.067	0.516	0.660±0.064	0.581	0.640±0.063	0.578

**Table 7:** AUROC values obtained in training (*mean* ± *standard deviation*) and in testing phases for each wavelet kernel with three ML models considered. Boldface values highlight the best three obtained results.

Wavelet Kernel	Machine Learning Model					
	XGB		SVM		RF	
	Train	Test	Train	Test	Train	Test
Bior1.5	0.685±0.045	0.652	0.725±0.044	0.689	0.711±0.047	0.706
Coif1	0.681±0.046	0.655	0.710±0.046	0.670	0.708±0.044	0.679
Db3	0.681±0.050	0.593	0.708±0.051	0.676	0.690±0.044	0.653
Dmey	0.684±0.052	0.611	0.700±0.049	0.650	0.678±0.047	0.662
Haar	0.710±0.048	0.636	0.734±0.047	0.677	0.726±0.046	0.686
Rbio1.5	0.674±0.049	0.623	0.700±0.050	0.649	0.697±0.047	0.649
Sym2	0.694±0.042	0.678	0.718±0.044	0.671	0.704±0.047	0.689
no wavelet	0.651±0.054	0.602	0.690±0.046	0.629	0.677±0.050	0.635

## Data Availability

Not applicable.

## Abbreviations

The following abbreviations are used in this manuscript:

AI	Artificial Intelligence
AUROC	Area Under Receiver Operating Characteristic
Bior	Biorthogonal Wavelet Kernel
Coif	Coiflets Wavelet Kernel
CT	Computed Tomography
CV	Cross Validation
CXR	Chest X-ray
Db	Daubechies Wavelet Kernel
Dmey	Discrete Meyer Wavelet kernel
DPI	Dots Per Inch
DWT	Discrete Wavelet Transform
FO	First Order
GLCM	Gray Level Co-occurrence Matrix
GLDM	Gray Level Dependence Matrix
GLRLM	Gray Level Run Length Matrix
GLSZM	Gray Level Size Zone Matrix
Haar	Haar Wavelet Kernel
LoG	Laplacian of Gaussian
MRI	Magnetic Resonance Imaging
NGTDM	Neighboring Gray Tone Difference Matrix
Rbio	Reverse Biorthogonal Wavelet Lernel
RF	Random Forest
ROI	Region Of Interest
SVM	Support Vector Machine
Sym	Symlets Wavelet kernel
XGB	XGBoost

## Appendix A

**Table A1:** Complete list of radiomic features selected via SFS, considering XGB model for each wavelet kernel considered.

Wavelet	Radiomic	Feature	Wavelet Kernel						
Decomposition	Category	Name	Bior1.5	Coif1	Db3	Dmey	Haar	Rbio1.5	Sym2
LL	FO	10Percentile	X	X		X	X	X	X
LL	FO	90Percentile	X	X			X	X	X
LL	FO	Kurtosis	X		X	X	X	X	
LL	FO	Minimum	X				X	X	
LL	FO	Range	X	X	X	X	X	X	X
LL	FO	Skewness	X	X	X	X	X	X	X
LL	GLRLM	GrayLevelNonUniformity			X		X		
LL	GLSZM	HighGrayLevelZoneEmphasis		X			X	X	X
LL	GLSZM	SmallAreaHighGrayLevelEmphasis		X	X		X	X	X
LH	FO	Energy	X	X			X		X
LH	FO	Kurtosis	X						
LH	FO	Skewness		X			X		X
LH	GLRLM	LongRunEmphasis			X			X	
LH	GLSZM	GrayLevelNonUniformity				X			
LH	GLSZM	HighGrayLevelZoneEmphasis	X				X		
LH	GLSZM	LargeAreaEmphasis				X			
LH	GLSZM	SizeZoneNonUniformity							
LH	GLSZM	SmallAreaHighGrayLevelEmphasis							
LH	GLSZM	ZoneEntropy			X		X		X
LH	GLDM	DependenceNonUniformity	X	X	X		X		X
LH	GLDM	DependenceVariance							X
LH	GLDM	LargeDependenceEmphasis		X		X	X	X	
LH	GLDM	LargeDependenceHighGrayLevelEmphasis							
HL	FO	Skewness		X					X
HL	FO	Maximum				X		X	
HL	GLRLM	LongRunEmphasis				X			
HL	GLSZM	HighGrayLevelZoneEmphasis	X						
HL	GLSZM	LargeAreaEmphasis	X				X		
HL	GLSZM	SizeZoneNonUniformity				X			
HL	GLSZM	SmallAreaHighGrayLevelEmphasis				X			
HL	GLSZM	ZoneEntropy	X	X	X		X	X	X
HL	GLDM	DependenceVariance							X
HH	FO	Minimum		X					X
HH	FO	Skewness							
HH	FO	Range				X			
HH	GLRLM	LongRunEmphasis							X
HH	GLRLM	LongRunHighGrayLevelEmphasis		X					
HH	GLSZM	GrayLevelNonUniformity	X		X				
HH	GLSZM	HighGrayLevelZoneEmphasis	X				X		
HH	GLSZM	SizeZoneNonUniformity				X		X	
HH	GLSZM	ZoneEntropy	X	X					
HH	GLDM	LargeDependenceHighGrayLevelEmphasis				X			
TOTAL SELECTED FEATURES			17	15	10	14	19	13	16

**Table A2:** Complete list of radiomic features selected via SFS, considering SVM model for each wavelet kernel considered.

Wavelet Decomposition	Radiomic Category	Feature Name	Wavelet Kernel						
			Bior1.5	Coif1	Db3	Dmey	Haar	Rbio1.5	Sym2
LL	FO	10Percentile	X			X	X	X	
LL	FO	90Percentile	X			X			
LL	FO	Kurtosis	X	X			X		X
LL	FO	Minimum		X	X			X	
LL	FO	Range			X	X	X	X	
LL	FO	Skewness	X	X	X	X	X	X	X
LL	GLRLM	GrayLevelNonUniformity						X	
LL	GLSZM	HighGrayLevelZoneEmphasis	X	X	X	X	X	X	
LL	GLSZM	SmallAreaHighGrayLevelEmphasis			X		X	X	
LH	FO	Energy					X		
LH	FO	Kurtosis	X				X		
LH	FO	Skewness	X				X		X
LH	GLRLM	LongRunEmphasis			X			X	
LH	GLSZM	GrayLevelNonUniformity				X			
LH	GLSZM	HighGrayLevelZoneEmphasis							
LH	GLSZM	LargeAreaEmphasis				X			
LH	GLSZM	SizeZoneNonUniformity							
LH	GLSZM	SmallAreaHighGrayLevelEmphasis							
LH	GLSZM	ZoneEntropy							
LH	GLDM	DependenceNonUniformity						X	
LH	GLDM	DependenceVariance		X					X
LH	GLDM	LargeDependenceEmphasis			X	X	X	X	
LH	GLDM	LargeDependenceHighGrayLevelEmphasis				X			
HL	FO	Kurtosis	X				X		
HL	FO	Skewness		X					X
HL	FO	Maximum				X		X	
HL	GLRLM	LongRunEmphasis			X	X			
HL	GLSZM	HighGrayLevelZoneEmphasis	X						
HL	GLSZM	LargeAreaEmphasis	X				X		
HL	GLSZM	SizeZoneNonUniformity				X			
HL	GLSZM	SmallAreaHighGrayLevelEmphasis							
HL	GLSZM	ZoneEntropy	X			X	X	X	
HL	GLDM	DependenceVariance							X
HH	FO	Skewness		X					
HH	FO	Range				X			
HH	GLRLM	LongRunEmphasis							X
HH	GLRLM	LongRunHighGrayLevelEmphasis		X	X				
HH	GLSZM	GrayLevelNonUniformity	X		X				
HH	GLSZM	HighGrayLevelZoneEmphasis	X				X		
HH	GLSZM	SizeZoneNonUniformity						X	
HH	GLSZM	ZoneEntropy	X				X		
HH	GLDM	LargeDependenceHighGrayLevelEmphasis				X			
TOTAL SELECTED FEATURES			14	9	10	15	15	13	8

**Table A3:** Complete list of radiomic features selected via SFS, considering RF model for each wavelet kernel considered.

Wavelet Decomposition	Radiomic Category	Feature Name	Wavelet Kernel						
			Bior1.5	Coif1	Db3	Dmey	Haar	Rbio1.5	Sym2
LL	FO	10Percentile			X	X	X		X
LL	FO	90Percentile			X	X		X	
LL	FO	Kurtosis	X	X	X		X	X	X
LL	FO	Minimum	X		X	X	X	X	X
LL	FO	Range	X		X		X		
LL	FO	Skewness	X	X	X	X	X	X	X
LL	GLRLM	GrayLevelNonUniformity			X		X		
LL	GLSZM	HighGrayLevelZoneEmphasis	X	X	X	X		X	
LL	GLSZM	SmallAreaHighGrayLevelEmphasis	X				X	X	
LH	FO	Energy							X
LH	FO	Kurtosis	X				X		
LH	FO	Skewness	X	X					X
LH	GLRLM	LongRunEmphasis			X				
LH	GLSZM	GrayLevelNonUniformity							
LH	GLSZM	HighGrayLevelZoneEmphasis	X			X	X		
LH	GLSZM	LargeAreaEmphasis							
LH	GLSZM	SizeZoneNonUniformity							
LH	GLSZM	SmallAreaHighGrayLevelEmphasis							
LH	GLSZM	ZoneEntropy		X			X		
LH	GLDM	DependenceNonUniformity		X		X			
LH	GLDM	DependenceVariance		X					X
LH	GLDM	LargeDependenceEmphasis	X		X	X	X	X	X
LH	GLDM	LargeDependenceHighGrayLevelEmphasis							
HL	FO	Kurtosis	X				X		
HL	FO	Skewness		X					X
HL	FO	Maximum				X		X	
HL	GLRLM	LongRunEmphasis							
HL	GLSZM	HighGrayLevelZoneEmphasis	X						
HL	GLSZM	LargeAreaEmphasis	X						
HL	GLSZM	SizeZoneNonUniformity				X			
HL	GLSZM	SmallAreaHighGrayLevelEmphasis				X			
HL	GLSZM	ZoneEntropy	X	X	X		X	X	X
HL	GLDM	DependenceVariance							
HH	FO	Minimum		X					X
HH	FO	Skewness		X					
HH	FO	Range				X			
HH	GLRLM	LongRunEmphasis							X
HH	GLRLM	LongRunHighGrayLevelEmphasis		X	X				
HH	GLSZM	GrayLevelNonUniformity			X		X		
HH	GLSZM	HighGrayLevelZoneEmphasis					X		
HH	GLSZM	SizeZoneNonUniformity						X	
HH	GLSZM	ZoneEntropy	X	X			X		
HH	GLDM	LargeDependenceHighGrayLevelEmphasis							
TOTAL SELECTED FEATURES			15	13	13	12	16	10	12
